# Noninvasive Neurally Adjusted Ventilation versus Nasal Continuous or Intermittent Positive Airway Pressure for Preterm Infants: A Systematic Review and Meta-Analysis

**DOI:** 10.3390/children10121935

**Published:** 2023-12-18

**Authors:** Mohammed Bhader, Mohammed Al-Hindi, Abdullah Ghaddaf, Anas Alamoudi, Amal Abualola, Renad Kalantan, Norah AlKhulifi, Ibrahim Halawani, Mansour Al-Qurashi

**Affiliations:** 1 College of Medicine, King Saud Bin Abdulaziz University for Health Sciences, Jeddah 21423, Saudi Arabia; alhindimy1@yahoo.com (M.A.-H.); abdullahg.official@gmail.com (A.G.); amalabualola.ksau@gmail.com (A.A.); renadktn1@gmail.com (R.K.); norah.alkhulifi@gmail.com (N.A.); qurashima@ngha.med.sa (M.A.-Q.); 2 King Abdullah International Medical Research Center, Jeddah 22384, Saudi Arabia; 3 Department of Pediatrics, King Abdulaziz Medical City, Jeddah 22384, Saudi Arabia; 4 College of Medicine, King Abdulaziz University, Jeddah 22252, Saudi Arabia; ibrahim1halawani@gmail.com

**Keywords:** preterm infants, noninvasive neurally adjusted ventilatory assist (NIV-NAVA), noninvasive ventilation, nasal continuous or positive airway pressure (NCPAP), intermittent positive airway pressure ventilation (NIPP), respiratory distress syndrome (RDS), systematic review

## Abstract

The noninvasive neurally adjusted ventilatory assist (NIV-NAVA) is a newly developed noninvasive ventilation technique with promising clinical and ventilatory outcomes for preterm infants. This systematic review and meta-analysis aimed to investigate whether NIV-NAVA has better clinical and ventilatory outcomes than nasal continuous airway pressure (NCPAP) or noninvasive positive pressure ventilation (NIPP) on premature infants. MEDLINE, Embase, and CENTRAL were searched, and randomized controlled trials (RCTs) that compared NIV-NAVA with NCPAP or NIPP for preterm infants (gestational age: <37 weeks) were included. We evaluated the following outcomes in the neonatal intensive care unit: the desaturation rate, failure of noninvasive modality requiring intubation when received as the primary mode or the need for re-intubation after extubation from mechanical ventilation in the secondary mode (weaning), length of stay, and fraction of inspired oxygen. The mean difference and risk ratio were used to represent continuous and dichotomous outcomes, respectively. We included nine RCTs involving 339 preterm infants overall. NIV-NAVA showed similar clinical and ventilatory outcomes to NCPAP or NIPP, except for the maximum diaphragmatic electrical activity. The rate of failure of the noninvasive modality was not statistically different between NIV-NAVA and NCPAP. The pooled estimates for the maximum electrical activity were significantly reduced in NIV-NAVA compared with those in NIPP. The findings suggest that NIV-NAVA may be as safe and effective as NCPAP and NIPP for preterm neonates, particularly those who may not tolerate these alternative noninvasive methods. However, further trials are recommended for greater evidence.

## 1. Introduction

Preterm birth is the leading cause of death worldwide in children <5 years. The World Health Organization defines prematurity in newborns as a birth occurring before 37 weeks of gestational age [1,2]. The global prevalence of preterm birth is approximately 10.6%, resulting in about 15 million live preterm births in 2014 [3]. Although the survival rate of premature infants has improved, further advances are vital to decreasing morbidity and mortality [4]. Respiratory distress syndrome (RDS) in neonates is triggered by low surfactant production and premature lungs. RDS is the leading reason for invasive ventilation in neonatal intensive care units (NICUs), although noninvasive measures are better in the form of lower ventilatory complications [5,6,7,8]. In addition to RDS, prematurity causes multiple adverse events, including bronchopulmonary dysplasia (BPD), sepsis, neurological conditions, and hearing and visual problems [9,10,11]. Although invasive ventilation and surfactant replacement can rescue newborns with RDS, the risks of BPD and lung injury may increase [12]. Hence, the European consensus recommends nasal continuous airway pressure (NCPAP) as the main modality for noninvasive ventilation in neonatal care as a primary mode [13]. Noninvasive positive pressure (NIPP) ventilation is an alternative treatment for managing RDS or to be used as a weaning mode post-extubation from mechanical ventilation [14]. NIPP may show more success in terms of prevention of post-extubation failure (re-intubation); however, it did not change the prevalence of BPD [15]. Moreover, NIPP may have a few disadvantages, including significant air leaks and the lack of synchronization with inspiratory efforts. In addition, the ventilator settings for NIPP are pressure-targeted, offering little adjustability for the variable ventilation demands of preterm neonates [16]. Noninvasive neutrally adjusted ventilatory assist (NIV-NAVA) is a recently developed mode of ventilation that utilizes the patient’s diaphragmatic electrical activity (Edi) to ensure better synchronization in and magnitude of the ventilation. Several electrodes installed on a modified intragastric feeding tube measure the Edi to determine diaphragmatic activity. The detection of infant diaphragmatic activity determines the beginning and end of inspiration to provide proportional and synchronized pressure support that depends on the newborns’ efforts. Infants ventilated using NIV-NAVA have better oxygenation and synchrony and tolerate extubation better [17,18,19,20,21,22]. Several randomized controlled trials (RCTs) compared the need for reintubation and clinical and ventilatory outcomes in infants after NIV-NAVA, NCPAP, and NIPP [23,24,25]. Additionally, two previous systematic reviews compared modality failure and other clinical and ventilatory outcomes after NIV-NAVA, NCPAP, and NIPP [26,27]. The present systematic review and meta-analysis include more studies and a larger sample size; moreover, this study was the first to produce results of the subgrouping analysis to compare NIV-NAVA to NCPAP and NIPP, depending on whether the ventilatory support was a primary or weaning modality. Here, clinical outcomes, ventilatory measures, and adverse effects are compared in infants ventilated using NIV-NAVA, NCPAP, or NIPP.

## 2. Study Design and Methods

The meta-analysis and systematic review were conducted following a prespecified protocol registered with PROSPERO (CRD42022342435). The report of this study was made according to the Preferred Reporting Items for Systematic Reviews and Meta-Analysis checklist [28].

### 2.1. Eligibility Criteria

RCTs, quasi-RCTs, cluster randomized trials, and crossover trials with the desired outcomes were included in the analysis. The crossover trials were incorporated into the meta-analysis using the methods described in the Cochrane Handbook for Systematic Reviews of Interventions [29]. The inclusion criteria were as follows: preterm infants of <37 weeks’ gestational age who received noninvasive respiratory support measures, including NIV-NAVA and NCPAP or NIPP ventilation, as primary or weaning (post-extubation from mechanical ventilation) respiratory support modes. Intervention period was any time from birth until discharge, except crossover trials; the ventilation was applied for at least 30 min. The studies reported at least one of the following outcomes: failure of noninvasive modality requiring intubation when received as primary mode or the need for re-intubation after extubation from mechanical ventilation in secondary mode (weaning), duration of oxygen supplementation, length of stay in the NICU, length of hospital stay, mean time to full enteral feeding, duration of invasive ventilation, heart rate (HR), respiratory rate (RR), need for surfactant therapy, desaturation, bradycardia, pneumothorax, apnea, patent ductus arteriosus (PDA), BPD, intraventricular hemorrhage (IVH), death, sepsis, necrotizing enterocolitis, retinopathy of prematurity, fraction of inspired oxygen (FiO_2_), oxygenation index, positive end-expiratory pressure (PEEP) or CPAP level (cmH_2_O), Edi including maximum Edi (μV), minimum Edi (μV), and swing Edi (μV), mean pH, mean pCO_2_, and mean airway pressure. Non-RCT studies and infants with congenital anomalies, neuromuscular disease, diaphragmatic paralysis, or palsy were excluded from the analysis. Refer to Section S1 for definitions and more information about the population, conditions, and ventilation modes included in this study.

### 2.2. Search Strategy

A systematic search was conducted using MEDLINE, Embase, and the Cochrane Central Register of Controlled Trials databases from the database inception till 26 June 2022, irrespective of the date or language. The search strategy is provided in the Supplementary Material. Refer to Section S2 for keywords used in the systematic search. Reference lists for the included RCTs were screened for related RCTs missed during the systematic search.

### 2.3. Study Selection and Data Extraction

The eligibility screening of titles and abstracts, full-text assessment, and data extraction from eligible trials were performed by two groups of reviewers independently and in duplicate, according to the prespecified selection criteria. Any disagreement was settled via consensus or discussion with separate field expert reviewers. The extracted data were entered into an Excel worksheet. The following data were extracted from each eligible trial: name of the first author and the year of publication, number of infants in each arm, gender, mean gestational age, mean birth weight, Apgar score at 5 min, premature rupture of membrane, cesarean delivery, and the clinical and ventilatory outcomes reported by each trial (e.g., failure of noninvasive modality requiring intubation when received as primary mode or the need for re-intubation after extubation from mechanical ventilation in weaning mode; length of stay in the NICU; duration of invasive ventilation; need for surfactant therapy; pneumothorax; Edi (μV), including maximum, minimum, and swing Edi; FiO_2_; PEEP; or CPAP level (cmH_2_O)).

### 2.4. Statistical Analysis

The meta-analysis was conducted using RevMan (Review Manager) version 5.3 (Cochrane Collaboration), employing a random-effects model. This study used a 95% confidence level and *p* < 0.05 as the threshold. Statistical heterogeneity was assessed using I2, and heterogeneity was assessed with the P of the Chi2 test. Continuous outcomes were represented as mean differences (MDs) and pooled using the inverse variance weighting method. Mean standardized differences (SMDs) were used for outcomes reported in different measuring units. Dichotomous outcomes were represented as risk ratios (RRs) and pooled using the inverse variance weighting method. Subgroup analyses were performed comparing NIV-NAVA, NCPAP, and NIPP based on whether ventilation was primary or weaning respiratory support. Those RCT findings that were excluded from the meta-analysis were reported narratively.

### 2.5. Risk of Bias Assessment

The risk of bias assessment for the RCTs was performed by two groups of reviewers independently using the Revised Cochrane Risk of Bias Assessment Tool in duplicate [30]. When 10 or more studies were included in a meta-analysis, publication bias was assessed by visual inspection of the funnel plot. This study utilized the Grading of Recommendations Assessment, Development, and Evaluation (GRADE) criteria to evaluate the certainty of the evidence for each outcome.

## 3. Results

**Figure 1:**
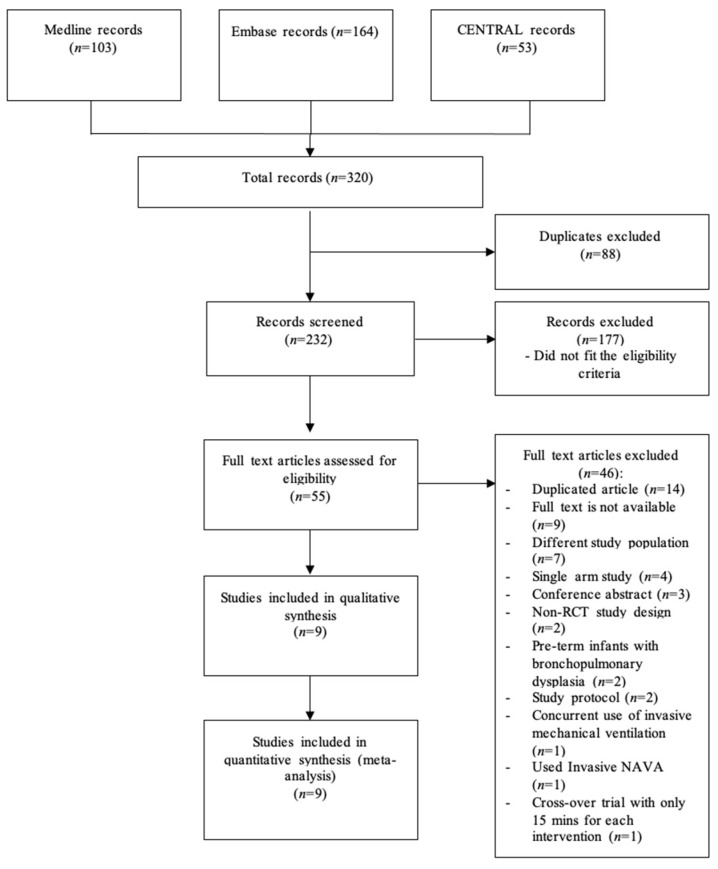
Study flow diagram.

The flowchart for the inclusion of studies in the review is shown in Figure 1. The literature search yielded 320 articles, and 88 duplicates were excluded. After screening the remaining 232 studies, 9 RCTs were considered eligible and included in the meta-analysis [18,23,24,25,31,32,33,34,35].

In total, 339 participants were included in this review. Sixty newborns were included in crossover trials [18,23,25,34]. Moreover, 197 (46.7%) newborns received NIV-NAVA, 152 (36%) received NCPAP, and 73 (17.3%) received NIPP. The numbers of males and females were 91 (48.2%) and 98 (51.8%) for participants who received NIV-NAVA, 85 (56%) and 67 (44%) for participants who received NCPAP, and 33 (50.7%) and 32 (49.3%) for participants who received NIPP. The gestational age (weeks) and the birth weight (g) in participants receiving NIV-NAVA, NCPAP, and NIPP are reported in Table 1. The studies’ characteristics and basic demographics are shown in Table 1.

The mode of ventilation was “primary intervention”, in which admitted premature infants with respiratory distress received noninvasive respiratory support as the initial intervention in three studies [24,31,35]. On the other hand, the intervention was labeled as a weaning intervention (post-extubation from mechanical ventilation) in six studies [18,23,25,32,33,34]. Four RCTs compared NIV-NAVA to NCPAP, four RCTs compared NIV-NAVA to NIPP, and one RCT compared NIV-NAVA to both NCPAP and NIPP.

**Figure 2:**
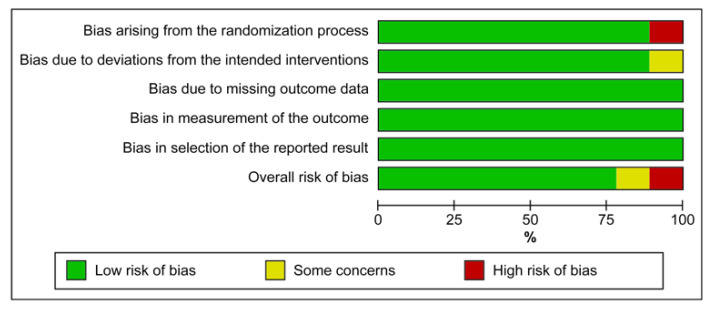
Risk of bias graph of the included studies.

**Figure 3:**
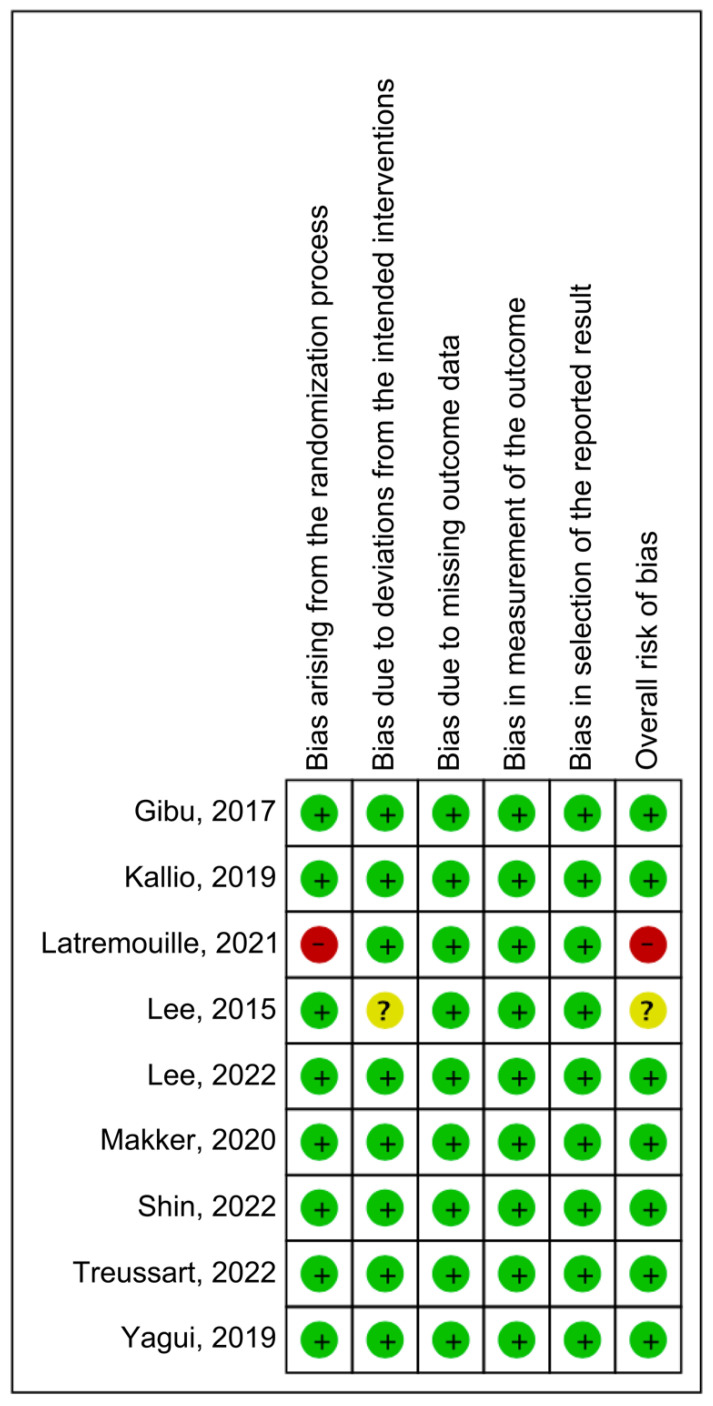
Risk of bias summary of the included studies [18,23,24,25,31,32,33,34,35].

Seven of the nine RCTs showed an overall low risk of bias. Some concerns and the source of bias being a deviation from the intended intervention were noted for one RCT. Furthermore, one RCT had an overall high risk of bias because of an issue in the randomization process domain (Figure 2 and Figure 3). Section S3 shows the Supplementary reported outcome analysis results (desaturation, bradycardia, PDA, IVH, time to full enteral feeding, pCO_2_, pH, RR, HR, and FiO_2_).

### 3.1. Noninvasive Modality Failure (Need for Intubation When Used as Primary Mode, and Need for Re-Intubation When Used as Weaning)

**Figure 4:**
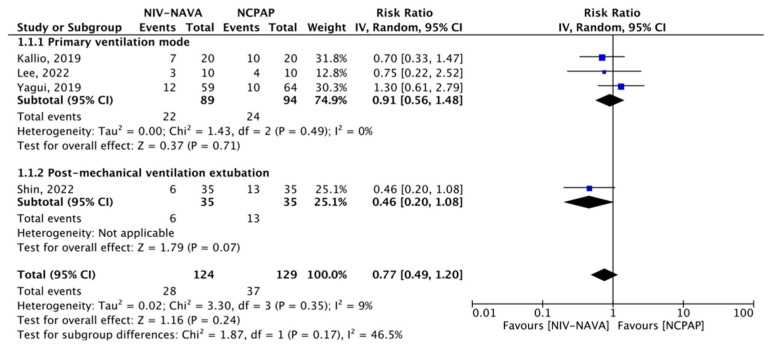
Forest plot of comparison between neurally adjusted ventilatory assist (NIV-NAVA) versus nasal continuous airway pressure (NCPAP), outcome “Noninvasive Modality failure (need for intubation when used as primary mode, and need for re-intubation when used as weaning)” [24,31,33,35].

Four of the RCTs reported the failure of noninvasive Modality (*n* = 253) [24,31,33,35]. The failure of noninvasive Modality was 28 (22.58%) and 37 (28.68%) in the NIV-NAVA and NCPAP groups, respectively, with no significant difference (RR = 0.77, 95% CI: 0.49 to 1.2, *p* = 0.24; I^2^ = 9%). In subgroup analyses based on whether the ventilation was provided as primary or weaning ventilatory support, no significant difference was noted (RR = 0.91, 95% CI: 0.56 to 1.48, *p* = 0.71; I^2^ = 0%; RR = 0.46, 95% CI: 0.20 to 1.02, *p* = 1.08; respectively) (Figure 4). The GRADE certainty of the evidence for the failure of the noninvasive modality was moderate; the grade was lowered by one level due to the imprecision noted in a few events or those that occurred because of participants (Table 2).

### 3.2. Need for Surfactant Therapy

**Figure 5:**
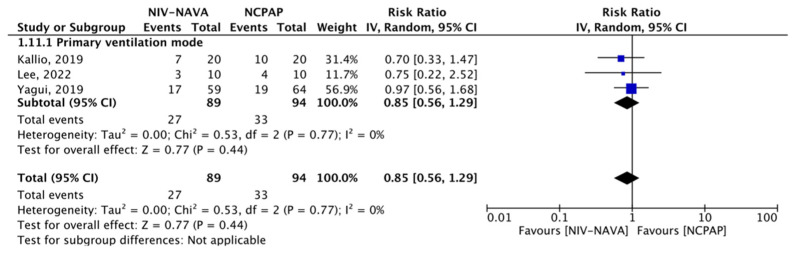
Comparison between NIV-NAVA and NCPAP; outcome: “Need for surfactant therapy” [24,31,35].

Five RCTs reported the need for surfactant therapy (*n* = 237) [24,31,32,34,35]. The need for surfactant therapy was 27 (30.33%) and 33 (35.1%) in the NIV-NAVA and NCPAP groups, respectively, and no significant difference was noted (RR = 0.85, 95% CI: 0.56 to 1.29, *p* = 0.44, I^2^ = 0%) (Figure 5). The GRADE certainty of the evidence was moderate for the need for surfactant therapy and was lowered by one level due to the imprecision noted in a few events or those that occurred because of participants (Table 2).

**Figure 6:**
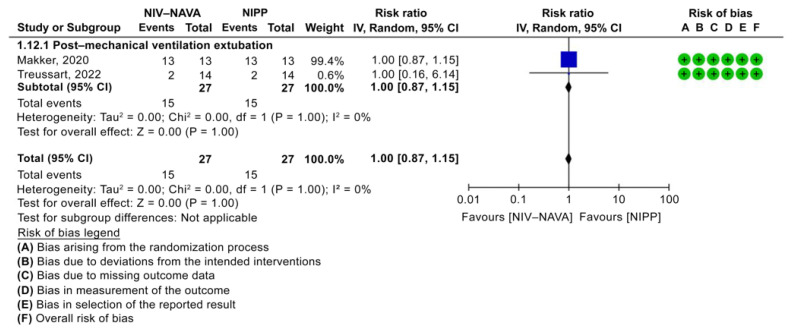
Forest plot of comparison between NIV-NAVA and NIPP; outcome: “Need for surfactant Therapy” [32,34].

The need for surfactant therapy was 15 (55.55%) and 15 (55.55%) in the NIV-NAVA and NIPP groups, respectively. No significant difference was noted between the two groups (RR = 1.00, 95% CI: 0.87 to 1.15, *p* = 1.00, I^2^ = 0%) (Figure 6). The evidence had moderate GRADE certainty for the need for surfactant therapy and was lowered by one level due to the imprecision noted in a few events or those that occurred because of participants (Table 2).

### 3.3. Pneumothorax

**Figure 7:**
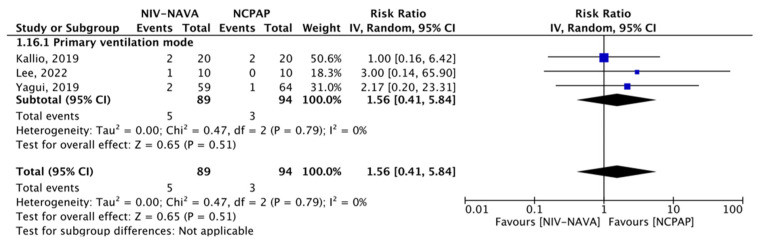
Forest plot of comparison between NIV-NAVA and NCPAP; outcome: “Pneumothorax” [24,31,35].

Three RCTs reported the incidence of pneumothorax (*n* = 183) [24,31,35]. Pneumothorax incidences were five (5.61%) and three (3.19%) in the NIV-NAVA and NCPAP groups, respectively, and no significant difference was detected between the NIV-NAVA and NCPAP groups (RR = 1.56, 95% CI: 0.41 to 5.84, *p* = 0.51, I^2^ = 0%) (Figure 7). The evidence had moderate GRADE certainty for pneumothorax and was lowered by one level due to the imprecision noted in a few events or those that occurred because of participants (Table 2). None of the post-extubation trials reported pneumothorax.

### 3.4. Bronchopulmonary Dysplasia

**Figure 8:**
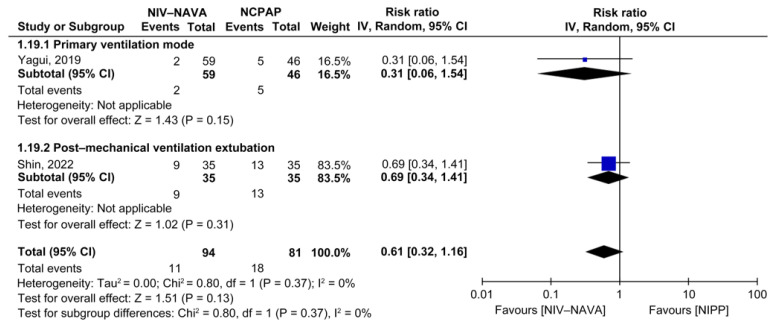
Forest plot of comparison between NIV-NAVA and NCPAP; outcome: “Bronchopulmonary dysplasia (BPD)” [33,35].

Two RCTs reported the incidence of BPD (*n* = 175) [31,33]. The numbers of BPDs were 11 (11.7%) and 18 (22.2%) in the NIV-NAVA and NCPAP groups, respectively, and no significant difference was detected between the two groups (RR = 0.61, 95% CI: 0.32 to 1.16, *p* = 0.13, I^2^ = 0%). Subgroup analysis comparing the NIV-NAVA and NCPAP groups based on whether the ventilatory support was primary or weaning showed no significant difference (RR = 0.31, 95% CI: 0.06 to 1.54, *p* = 0.15; RR = 0.69, 95% CI: 0.34 to 1.41, *p* = 0.31, respectively) (Figure 8). The evidence had moderate GRADE certainty for BPD and was lowered by one level due to the imprecision noted in a few events or those that occurred because of participants (Table 2).

### 3.5. Length of NICU Stay

Only one RCT reported the length of stay in the NICU (*n* = 40) [24]. The NICU length of stay means were 15.9 days and 16.2 days in the NIV-NAVA and NCPAP groups, respectively, and no significant difference was noted between the groups (MD = −0.3, 95% CI: −6.93 to 6.33, *p* = 0.93). A calculation of the heterogeneity (I^2^) was not feasible.

### 3.6. Length of Hospital Stay

**Figure 9:**
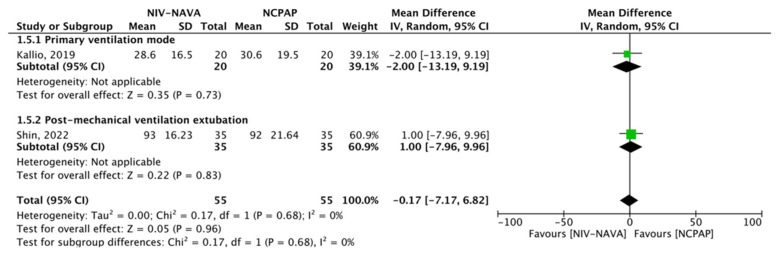
Forest plot of comparison between NIV-NAVA and NCPAP; outcome: “Length of hospital stay” [24,33].

Two RCTs reported the length of hospital stays (*n* = 110) [24,33]. The weighted averages of the length of hospital stays were 67.81 days and 67.99 days in the NIV-NAVA and NCPAP groups, respectively, and no significant difference was noted (MD = −0.17, 95% CI: −7.17 to 6.82, *p* = 0.96, I^2^ = 0%). A subgroup analysis comparing the NIV-NAVA and NCPAP groups based on whether the ventilatory support was primary or weaning showed no significant difference (RR = −2.00, 95% CI: −13.19 to 9.19, *p* = 0.73; RR = 1.00, 95% CI: −7.96 to 9.96, *p* = 0.83, respectively) (Figure 9). The GRADE certainty of the evidence was rated moderate for the length of hospital stays and was lowered by 1 due to the imprecision noted in a few events or those that occurred because of participants (Table 2).

### 3.7. Duration of Invasive Ventilation

**Figure 10:**
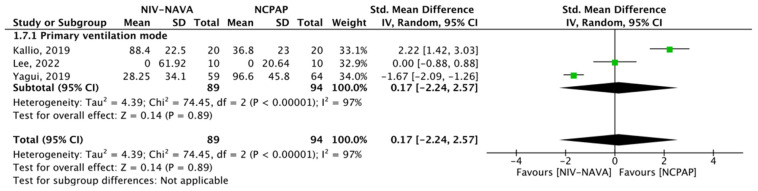
Forest plot of comparison between NIV-NAVA and NCPAP; outcome: “Duration of invasive ventilation” [24,31,35].

Three RCTs reported the duration of invasive ventilation (*n* = 183) [24,31,35]. The weighted average durations of invasive ventilation were 38.86 and 45.02 days in the NIV-NAVA and NCPAP groups, respectively. No significant difference was noted between the two groups (SMD = 0.17, 95% CI: −2.24 to 2.57, *p* = 0.89, I^2^ = 97) (Figure 10). The GRADE certainty of evidence was low for the duration of invasive ventilation and was lowered by two levels because of the imprecision and inconsistency given the few participants and the considerable statistical heterogenicity (Table 2).

### 3.8. Electrical Activity of the Diaphragm (Edi)

Three RCTs reported the maximum Edi (*n* = 104) [18,25,34]. Only one study compared NIV-NAVA to NCPAP [24]. The mean maximum Edis were 58.2 and 62.5 in the NIV-NAVA and NCPAP groups, respectively, and no significant difference was noted between the groups (MD = −4.30, 95% CI: −25.98 to 17.38, *p* = 0.70). The calculation of heterogeneity (I2) was not feasible.

**Figure 11:**
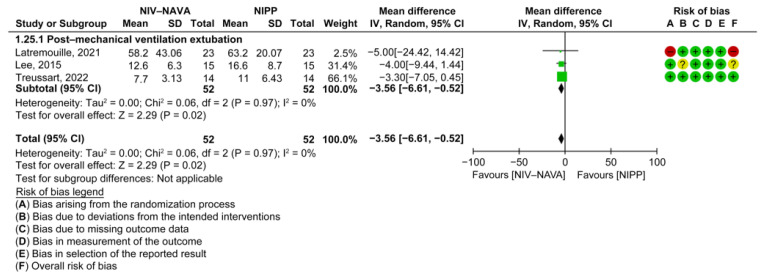
Forest plot of comparison between NIV-NAVA and NIPP; outcome: “Maximum Edi (uV)” [18,25,34].

The weighted averages of maximum Edis were 10.5 and 14 in the NIV-NAVA and NIPP groups, respectively, and a significant difference was noted between the groups (MD = −3.56, 95% CI: −6.61 to −0.52, *p* = 0.02, I^2^ = 0%) (Figure 11). The GRADE certainty of the evidence was low for the maximum Edi when comparing NIV-NAVA to NIPP and was lowered by two levels because of the imprecision and risk of bias (Table 2).

Only one study compared minimum Edis between the NIV-NAVA and NCPAP groups [25]. The mean minimum Edis were 0 and 0 in the NIV-NAVA and NCPAP groups, respectively, and no significant difference was noted between the groups (MD = 0.00, 95% CI: −0.03 to 0.03, *p* = 1.00). The calculation of heterogeneity (I^2^) was not feasible.

**Figure 12:**
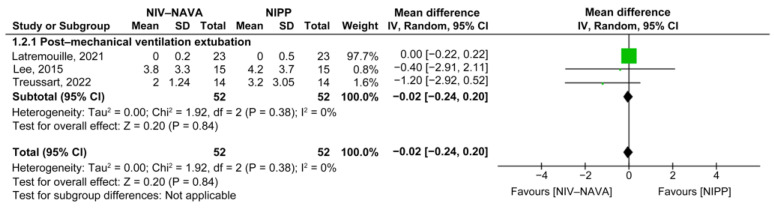
Forest plot of comparison between NIV-NAVA and NIPP; outcome: “Minimum Edi (uV)” [18,25,34].

The weighted average minimum Edis were 0.06 and 0.08 in the NIV-NAVA and NIPP groups, respectively, and no significant difference was noted between the groups (MD = −0.02, 95% CI: −0.24 to 0.20, *p* = 0.84, I^2^ = 0%) (Figure 12). The GRADE certainty of evidence was low for maximum Edi when comparing NIV-NAVA to NIPP and was lowered by two levels because of the imprecision and risk of bias (Table 2).

**Figure 13:**
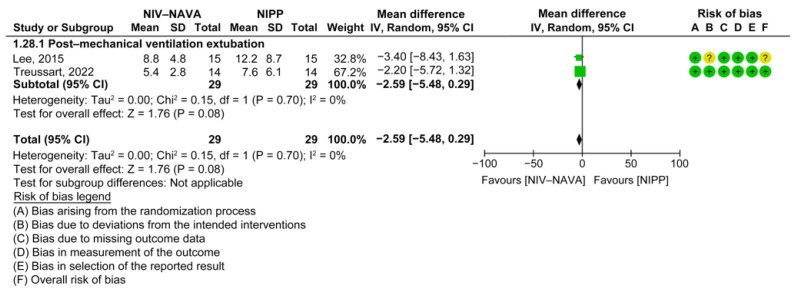
Forest plot of comparison between NIV-NAVA and NIPP; outcome: “Swing Edi (uV)” [18,34].

Two RCTs reported swing Edis (*n* = 58) [18,34]. The weighted average swing Edis were 6.51 and 9.1 in the NIV-NAVA and NIPP groups, respectively. No significant difference was noted between the two groups (MD = −2.59, 95% CI: −5.48 to 0.29, *p* = 0.08, I^2^ = 0%) (Figure 13). For the swing Edi, the evidence had a moderate GRADE certainty and was lowered by one level because of the imprecision (Table 2).

### 3.9. Other Outcomes

No statistical significance was noted in the desaturation, bradycardia, PDA, IVH, time to full enteral feeding, pCO_2_, pH, RR, HR, and FiO_2_ between NIV-NAVA and NCPAP or NIPP. Refer to Section S3 in the Supplementary Material for a detailed analysis and figures.

## 4. Discussion

In this systematic review and meta-analysis, the pooled estimates were significantly reduced for the maximum Edi in favor of NIV-NAVA compared to NIPP. However, no significant differences in other ventilatory, clinical, and adverse outcomes were found between the NIV-NAVA and NCPAP or NIPP groups or in the subgroup analyses based on whether the ventilatory support was the primary or weaning modalities.

The failure of the noninvasive modality (need for intubation when used as a primary mode or need for re-intubation when used as weaning) is a commonly reported key performance indicator, and the prevention of intubation/re-intubation can reduce adverse outcomes. For the primary mode, the consensus recommends a noninvasive approach as a primary rescue for preterm infants with evidence of respiratory distress. The failure of such a method results in intubation and surfactant administration and is inversely related to the degree of prematurity and accounts for 50% of preterm infants weighing <1500 g [36,37,38].

In a meta-analysis, if applied within six hours after birth, NIPP reduces the risk of respiratory failure and the need for intubation in very preterm infants (GA 28 weeks and above) with a risk for RDS. It may also decrease the rate of BPD slightly [39]. In this meta-analysis, there was no significant difference between the NIV-NAVA and NCPAP in terms of failure for the noninvasive modality (requiring intubation) as a primary modality.

Systematic reviews proved the superiority of NIPP ventilation over NCPAP as a weaning mode post-extubation from mechanical ventilation in preterm infants in regards to extubation failure (re-intubation). There is a paucity of data for infants less than 28 weeks gestation [15]. A recent RCT comparing the success rate of elective extubation between NIV-NAVA and NIPP demonstrated no statistical reduction in the risk of extubation failure [32]. Nevertheless, another RCT reported the superiority of NIV-NAVA to NCPAP in reducing extubation failure, which is consistent with two previous retrospective studies [33,40,41]. In this meta-analysis, the rate of mechanical reintubation after post-extubation was not significantly different between NIV-NAVA and NCPAP. Moreover, in this study, the trials included more extreme preterm infants than Lemyre et al. found [15]. Our conclusion is consistent with a recent Cochrane review [26].

Notably, the trials that were carried out to investigate the primary modes included higher gestational ages and birth weights than the trials for the post-extubation; see Table 1. This was similar to the conclusion of another meta-analysis that compared NIPP to NCAP. However, most trials enrolled infants with a gestational age of very preterm infants from 28 to 32 weeks, with an overall mean gestational age of around 30 weeks. As such, the results of this review may not apply to extremely preterm infants (<28 weeks) who are most at risk of needing mechanical ventilation or developing BPD. Additional studies are needed to confirm these results and to assess the safety of NIPP compared with NCPAP alone in a larger patient population [40].

According to the European Consensus Guidelines on the Management of Respiratory Distress Syndrome, multiple multicenter RCTs demonstrated that the early use of surfactants in preterm infants resulted in a lower incidence of BPD and pneumothorax [13]. However, in our study, no significant differences in surfactant therapy, incidences of BPD, and pneumothorax were detected between the ventilation modalities. A recent meta-analysis comparing NIV-NAVA to NCPAP demonstrated similar results [27]. Moreover, multiple RCTs reported insignificant differences between NIV-NAVA and the usual noninvasive care [18,24,27,31,32,33,35]. For the duration of the invasive ventilation, there was a wide heterogeneity between the studies. Hence, future multicentric studies with a more real-world approach are needed to delineate this issue.

Here, preterm infant electrical diaphragm activity between NIV-NAVA and NCPAP or NIPP was compared. Electrical diaphragm activity was measured as maximum, minimum, and swing Edi. Although the results demonstrated no significant differences in minimum and swing Edi, maximum Edi favored NIV-NAVA to NIPP. Acquiring a lower maximum Edi would favor NIV-NAVA in terms of better breathing and synchronicity. However, this is a short-term advantage. This was not reflected in any clinical or short-term benefits in this or earlier meta-analyses (Cochrane). Trials measuring long-term outcomes are crucial to further determine which ventilatory modality is superior.

In comparison to the previous Cochrane review, Goel et al., our study produced results by analyzing desaturation, bradycardia, PDA, IVH, time to full enteral feeding, pCO_2_, pH, and HR [26].

To the best of our knowledge, only two systematic reviews and meta-analyses compared NIV-NAVA to other noninvasive modalities in terms of the failure of the noninvasive modality requiring intubation when used as a primary mode and the need for re-intubation when used as weaning, as well as other clinical and ventilatory outcomes [26,27]. Our meta-analysis is the first to produce results from the subgroup analyses comparing ventilation modalities used as primary or weaning interventions. Moreover, this is the first meta-analysis comparing swing Edi and involves a larger sample size compared to other reviews from high-level evidence RCTs. In our review, most of the RCTs were well-conducted and showed an overall low risk of bias, thus putting forward that RCT findings were consistent, had a low rate of heterogeneity, and provided optimal evidence for meta-analysis. Nonetheless, we are aware that our review has substantial limitations. The assessment of publication bias was not feasible since no outcome was reported in more than 10 studies. Most of the included studies focused on short-term ventilatory and clinical outcomes. Thus, we recommend more trials measuring long-term outcomes, including healthcare utilization and pulmonary and neurodevelopmental outcomes [42,43,44]. Most of the eligible RCTs were single-center studies with relatively low sample sizes. Multicenter trials with extended risk factor analyses, such as maternal risk scores and multidomain socioeconomic status, are recommended to obtain greater evidence [45,46].

## 5. Conclusions

This systematic review and meta-analysis compared NIV-NAVA with NCPAP or NIPP for preterm infants with RDS. A significant difference in maximum Edi favored NIV-NAVA compared to NIPP. No significant differences in other clinical and ventilatory outcomes were detected between NIV-NAVA and NCPAP or NIPP. No significant difference was found in subgroup analyses between NIV-NAVA, NCPAP, and NIPP based on whether ventilatory support was given as a primary or weaning modality. Further trials are recommended to obtain greater evidence.

## Figures and Tables

**Table 1:** Studies’ characteristics [18,23,24,25,31,32,33,34,35].

	Study, Year (Sample Size) (Type of Trial)	Gender			Mean Gestational Age (Weeks)			Mean Birth Weight (g)		
		NIV-NAVA	NCPAP	NIPP	NIV-NAVA	NCPAP	NIPP	NIV-NAVA	NCPAP	NIPP
		M/F	M/F	M/F						
Primary Mode	Kallio, 2019 (*n* = 40) (RCT)	1:1	1.86:1	-	33.1 (±2.0) *	33.0 (±1.8) *	-	2140 (±766) *	2122 (±776) *	-
	Lee, 2022 (*n* = 10) (RCT)	1:1.5	1:1.5	-	29.57 (±2) *	29.9 (±1.29) *	-	1331 (±370) *	1346 (±379) *	-
	Yagui, 2019 (*n* = 123) (RCT)	1:1.68	1.13:1	-	29.6 (±2.1) *	29.8 (±2.1) *	-	1077.8 (±259) *	1130 (±258.4) *	-
Secondary (weaning) Mode	Shin, 2022 (*n* = 70) (RCT)	1.5:1	1.9:1	-	26.6 (25.4–28.3) **	27.1 (26–29) **	-	880 (740–1110) **	970 (740–1120) **	-
	Lee, 2015 (*n* = 15) (C/O)	1.5:1	-	1.5:1	27.1 (26–28.29) ***	-	27.1 (26–28.29) ***	790 (675–1215) ***	-	790 (675–1215) ***
	Treussart, 2022 (*n* = 14) (C/O)	1.3:1	-	1.3:1	25.6 (25.3–26.4) **	-	25.6 (25.3–26.4) **	755 (686–824) **	-	755 (686–824) **
	Makker, 2020 (*n* = 26) (RCT)	1:1.17	-	1:1.6	27 (25–28) **	-	27 (26–30) **	1000 (840–1120) **	-	990 (690–1370) **
	Gibu, 2017 (*n* = 8) (C/O)	-	-	-	25.48 (±1.48) *	-	25.48 (±1.48) *	791.125 (±209.35) *	-	791.125 (±209.35) *
	Latremouille, 2021 (*n* = 23) (C/O)	1:1.09	1:1.09	1:1.09	25.9 (25.2–26.4) **			760 (595–900) **		

NIV-NAVA: noninvasive neurally adjusted ventilatory assist, NIPP: nasal intermittent positive airway pressure, NCPAP: nasal continuous or positive airway pressure, C/O: cross-over study, *: mean (SD), **: median (IQR), ***: mean (range).

**Table 2:** Grading of development, recommendations assessment, and evaluation criteria.

Outcome (Comparison)	Certainty Assessment						
	Participants (Studies)	Risk of Bias	Inconsistency	Indirectness	Imprecision	Publication Bias	Overall Certainty of Evidence
Modality Failure (NIV-NAVA VS. NCPAP)	253 (4 RCTs)	not serious	not serious	not serious	serious ^a^	none	Moderate
Length of hospital stay (NIV-NAVA VS. NCPAP)	110 (2 RCTs)	not serious	not serious	not serious	serious ^a^	none	Moderate
Mean time to full enteral feeding (NIV-NAVA VS. NCPAP)	110 (2 RCTs)	not serious	not serious	not serious	serious ^b^	none	Moderate
Duration of invasive ventilation after intubation (NIV-NAVA VS. NCPAP)	183 (3 RCTs)	not serious	serious ^c^	not serious	serious ^a^	none	Low
Need for surfactant therapy (NIV-NAVA VS. NCPAP)	183 (3 RCTs)	not serious	not serious	not serious	serious ^a^	none	Moderate
Need for surfactant therapy (NIV-NAVA VS. NIPP)	54 (2 RCTs)	not serious	not serious	not serious	serious ^a^	none	Moderate
Desaturation (NIV-NAVA VS. NCPAP)	116 (2 RCTs)	serious ^b^	not serious	not serious	serious ^a^	none	Low
Desaturation (NIV-NAVA VS. NIPP)	76 (2 RCTs)	serious ^b^	serious ^d^	not serious	serious ^a^	none	Very low
Bradycardia (NIV-NAVA VS. NIPP)	76 (2 RCTs)	serious ^b^	not serious	not serious	serious ^a^	none	Low
Pneumothorax (NIV-NAVA VS. NCPAP)	183 (3 RCTs)	not serious	not serious	not serious	serious ^a^	none	Moderate
Apnea (NIV-NAVA VS. NCPAP)	193 (2 RCTs)	not serious	serious ^d^	not serious	serious ^a^	none	Low
Patent ductus arteriosus (NIV-NAVA VS. NCPAP)	175 (2 RCTs)	not serious	serious ^d^	not serious	serious ^a^	none	Low
BPD (NIV-NAVA VS. NCPAP)	175 (2 RCTs)	not serious	not serious	not serious	serious ^a^	none	Moderate
Intraventricular hemorrhage (NIV-NAVA VS. NCPAP)	215 (3 RCTs)	not serious	not serious	not serious	serious ^a^	none	Moderate
FiO_2_ (NIV-NAVA VS. NCPAP)	110 (2 RCTs)	not serious	not serious	not serious	serious ^a^	none	Moderate
FiO_2_ (NIV-NAVA VS. NIPP)	88 (3 RCTs)	not serious	very serious ^c^	not serious	serious ^a^	none	Very low
Respiratory rate (NIV-NAVA VS. NIPP)	44 (2 RCTs)	not serious	not serious	not serious	serious ^a^	none	Moderate
maximum Edi (NIV-NAVA VS. NIPP)	104 (3 RCTs)	serious ^b^	not serious	not serious	serious ^a^	none	Low
swing Edi (NIV-NAVA VS. NIPP)	58 (2 RCTs)	not serious	not serious	not serious	very serious ^a^	none	Low
minimum Edi (NIV-NAVA VS. NIPP)	104 (3 RCTs)	serious ^b^	not serious	not serious	serious ^a^	none	Low
Mean pH NIV-NAVA VS. NCPAP)	110 (2 RCTs)	not serious	not serious	not serious	serious ^a^	none	Moderate
Mean pCO_2_ (NIV-NAVA VS. NCPAP)	110 (2 RCTs)	not serious	not serious	not serious	serious ^a^	none	Moderate

^a^. Small sample size or wide 95% CI; ^b^. Some concerns or high risk of bias; ^c^. Considerable statistical heterogeneity; ^d^. Substantial statistical heterogeneity.

## Data Availability

The data presented in this study are available in article.

## Supplementary Materials

The following supporting information can be downloaded at: https://www.mdpi.com/article/10.3390/children10121935/s1, Supplementary Section S1: Definitions and more information about the population, conditions, and ventilation modes included in this study. Supplementary Section S2: Search strategy. Section S3: Supplementary Outcomes. Figure S1: Forest plot of comparison between NIV–NAVA and NCPAP; outcome: “Desaturation”. Figure S2: Forest plot of comparison between NIV–NAVA and NIPP; outcome: “Desaturation”. Figure S3: Forest plot of comparison between NIV–NAVA and NIPP; outcome: “Bradycardia”. Figure S4: Forest plot of comparison between NIV–NAVA and NCPAP; outcome: “Apnea”. Figure S5: Forest plot of comparison between NIV–NAVA and NCPAP; outcome: “Patent ductus arteriosus (PDA)”. Figure S6: Forest plot of comparison between NIV–NAVA and NCPAP; outcome: “Intraventricular hemorrhage (IVH)”. Figure S7: Forest plot of comparison between NIV–NAVA and NCPAP; outcome: “Mean time to full enteral feeding (days)”. Figure S8: Forest plot of comparison between NIV–NAVA and NCPAP; outcome: “Mean pCO_2_”. Figure S9: Forest plot of comparison between NIV–NAVA and NCPAP; outcome: “Mean pH”. Figure S10: Forest plot of comparison between NIV–NAVA and NIPP; outcome: “Respiratory rate (RR)”. Figure S11: Forest plot of comparison between NIV–NAVA and NCPAP; outcome: “Fraction of inspired oxygen (FiO_2_)”. Figure S12: Forest plot of comparison between NIV–NAVA and NIPP; outcome: “Fraction of inspired oxygen (FiO_2_)”. Refs. [47,48] are cited in the Supplementary Materials.
