# Supplementary material for: Noninvasive Neurally Adjusted Ventilation versus Nasal Continuous or Intermittent Positive Airway Pressure for Preterm Infants: A Systematic Review and Meta-Analysis
Source: Children (Basel). 2023 Dec 18;10(12):1935. doi: 10.3390/children10121935 (PMC10741611; doi:10.3390/children10121935)
Supplement: Supplementary file 1 [file children-10-01935-s001.zip › children-2669063-supplementary.pdf]

Section S1

About the **population**:

According to the criteria developed in 2005 by the National Institute of Child Health and Human Development Workshop, preterm Infants are those who were born with a gestational age of less than 37 weeks. [1]

About the **condition**:

The incidence of respiratory distress syndrome (RDS) in premature infant is proportionally increased as the gestational age of the baby decreased. [2] A preterm infant could experience apnea due to the immature development of the lung and reduced production of surfactant; moreover, the reduced of rib cage compliance, floppy airways, and immature responsiveness of respiratory centers and chemoreceptors are additional etiologies to the respiratory failure. [3]

About the **intervention**:

As opposed to any other method of support that relied on pressure to deliver non-invasive respiratory support, NIV-NAVA utilized electrical diaphragm activity to initiate and assist inspiratory effort. Any non-invasive respiratory support system that continuously maintains positive end expiratory pressure (> +1 cmH2O) with inspiratory support activated by electrical diaphragmatic activity and detected by transesophageal, subcutaneous, or transcutaneous sensors was considered NIV-NAVA in our study. [4]

About the **Ventilation** modes:

NCPAP

Regardless of flow rate or oxygen need, we defined non-invasive continuous positive end expiratory pressure at a specified pressure larger than +1 cmH2O as NCPAP.

NIPP

Non-invasive continuous positive end expiratory pressure (NIPPV) was defined as continuous positive end expiratory pressure (nCPAP) with any extra inspiratory support, either not synchronized with breathing or activated by mechanisms other than electrical diaphragm activity.

Section S2

The following keywords were used for the systematic search:

1. exp Obstetric Labor, Premature/ or exp Premature Birth/ or exp Infant, Premature/ or exp Infant, Newborn/
2. Premature$.mp.
3. Infant$.mp.
4. Newborn$.mp.
5. exp Respiratory Distress Syndrome/
6. Respiratory Distress Syndrome$.mp.
7. Respiratory Distress$.mp.
8. exp Birth Weight/
9. Low birth weight$.mp.
10. Neonatal lung disease$.mp.
11. exp Intubation, Intratracheal/ or exp Intubation/
12. Intubation$.mp.

13. Mechanical ventilation$.mp.

14. exp Ventilator Weaning/

15. Ventilator weaning$.mp.

16. 1 or 2 or 3 or 4 or 5 or 6 or 7 or 8 or 9 or 10 or 11 or 12 or 13 or 14 or 15

17. Neurally adjusted ventilation$.mp.

18. Neurally adjusted ventilatory assist$.mp.

19. 12 or 13

20. Respiration, Artificial / or exp Noninvasive Ventilation/

21. Noninvasive ventilation$.mp.

22. exp Interactive Ventilatory Support/

23. exp Positive-Pressure Respiration/ or exp Continuous Positive Airway Pressure/

24. Continuous positive airway pressure$.mp.

25. exp Intermittent Positive-Pressure Ventilation/

26. Intermittent positive-pressure ventilation$.mp.

27. 15 or 16 or 17 or 18 or 19 or 20 or 21

28. exp Randomized Controlled Trial/ or exp Clinical Trial/

29. Trial$.mp.

30. 23 or 24

31. 16 and 19 and 27 and 30

Section S3: Supplementary Outcomes

Supplementary Figure S1: Comparison Between NIV- NAVA Versus NCPAP, outcome "Desaturation"

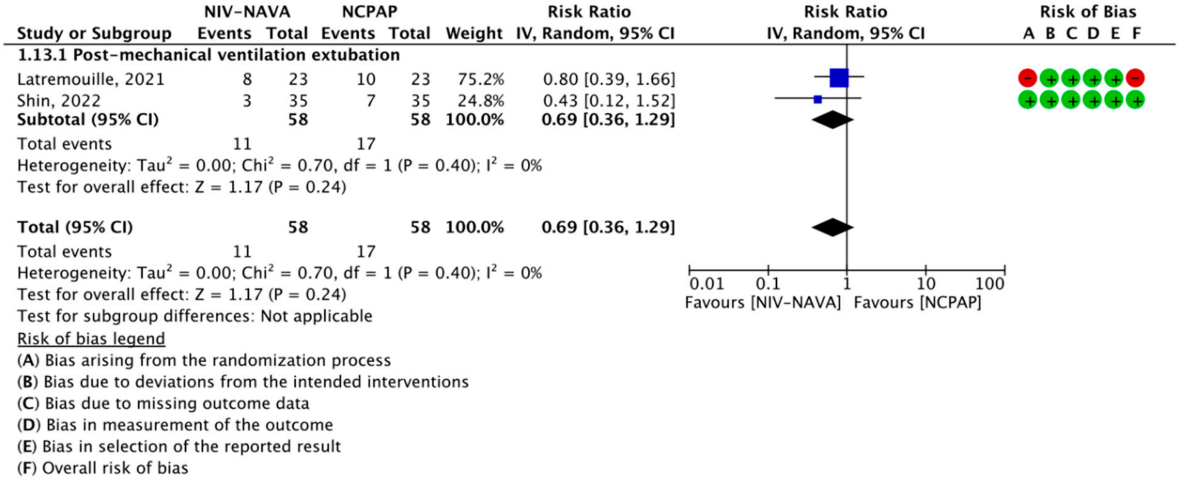

Supplementary Figure S2: Comparison Between NIV- NAVA Versus NIPP, outcome "Desaturation"

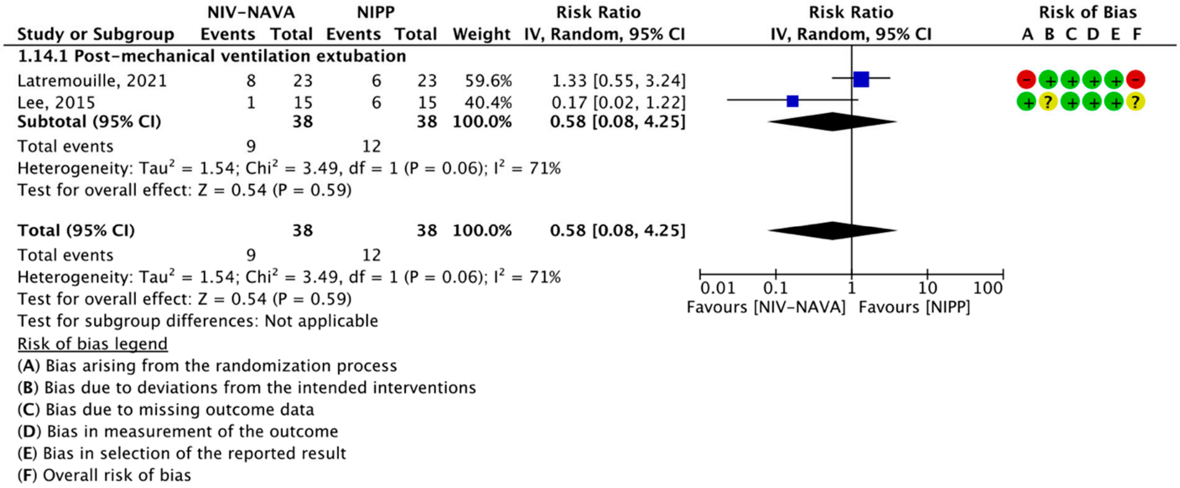

Supplementary Figure S3: Comparison Between NIV- NAVA Versus NIPP, outcome "Bradycardia"

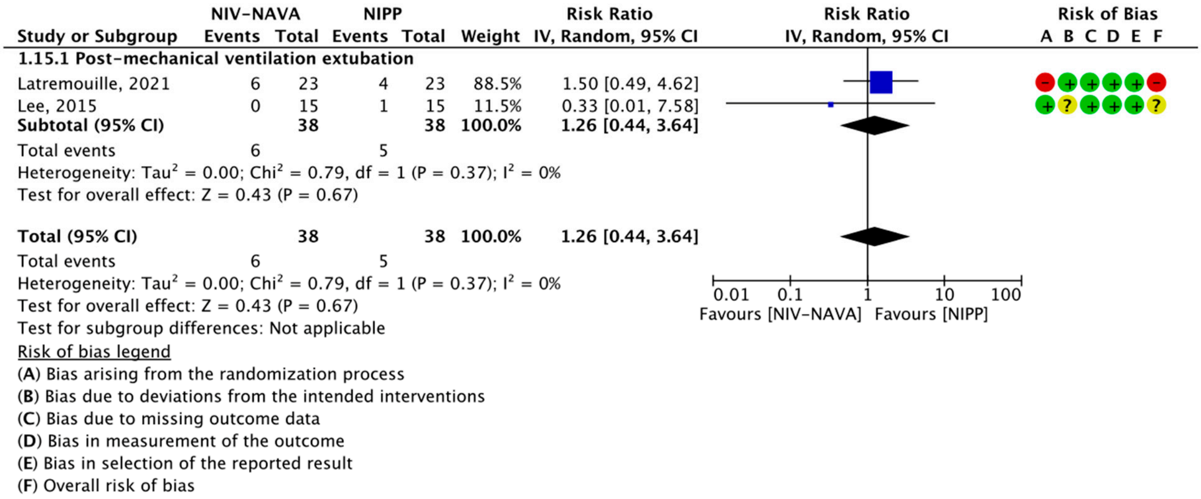

Supplementary Figure S4: Comparison Between NIV- NAVA Versus NCPAP, outcome "Apnea"

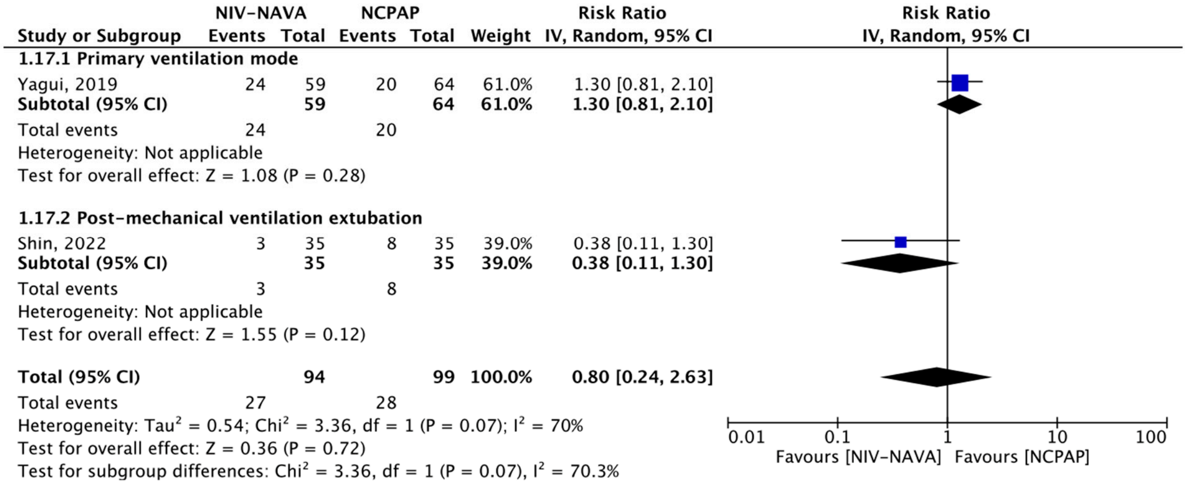

Supplementary Figure S5: Comparison Between NIV- NAVA Versus NCPAP, outcome "Patent ductus arteriosus (PDA)"

Study or Subgroup	NIV-NAVA Events	NIV-NAVA Total	NCPAP Events	NCPAP Total	Weight	Risk Ratio IV, Random, 95% CI
**1.18.1 Primary ventilation mode**						
Yagui, 2019	15	59	17	46	16.7%	0.69 [0.39, 1.23]
**Subtotal (95% CI)**		**59**		**46**	**16.7%**	**0.69 [0.39, 1.23]**
Total events	15		17			
Heterogeneity: Not applicable						
Test for overall effect: Z = 1.27 (P = 0.20)						
**1.18.2 Post-mechanical ventilation extubation**						
Shin, 2022	25	35	29	35	83.3%	0.86 [0.67, 1.12]
**Subtotal (95% CI)**		**35**		**35**	**83.3%**	**0.86 [0.67, 1.12]**
Total events	25		29			
Heterogeneity: Not applicable						
Test for overall effect: Z = 1.13 (P = 0.26)						
**Total (95% CI)**		**94**		**81**	**100.0%**	**0.83 [0.66, 1.05]**
Total events	40		46			
Heterogeneity: Tau² = 0.00; Chi² = 0.49, df = 1 (P = 0.48); I² = 0%						
Test for overall effect: Z = 1.55 (P = 0.12)						
Test for subgroup differences: Chi² = 0.49, df = 1 (P = 0.48), I² = 0%						

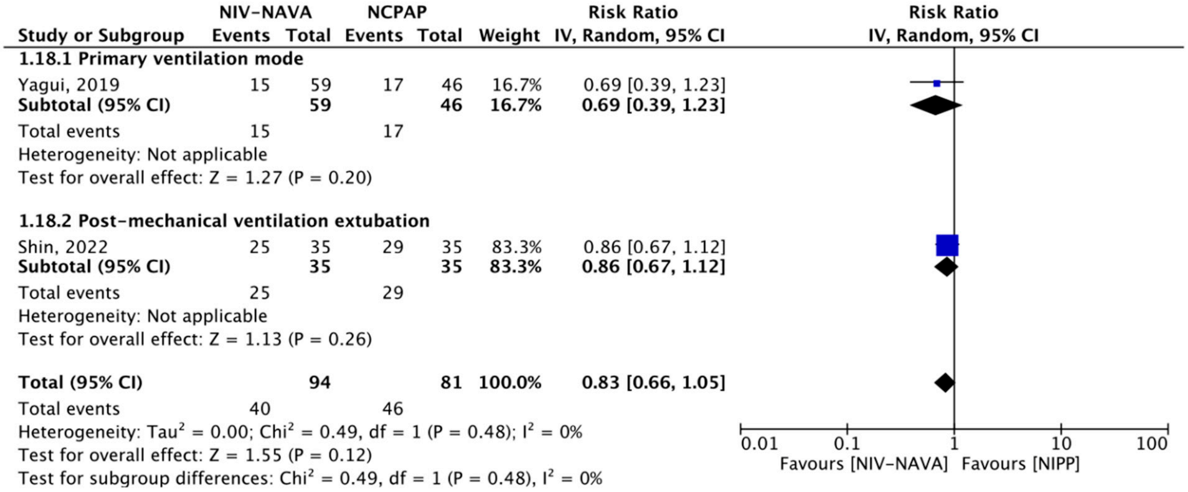

Supplementary Figure S6: Comparison Between NIV- NAVA Versus NCPAP, outcome "Intraventricular hemorrhage (IVH)"

Study or Subgroup	NIV-NAVA Events	NIV-NAVA Total	NCPAP Events	NCPAP Total	Weight	Risk Ratio IV, Random, 95% CI
**1.20.1 Primary ventilation mode**						
Kallio, 2019	1	20	0	20	5.6%	3.00 [0.13, 69.52]
Yagui, 2019	11	59	7	46	73.7%	1.23 [0.52, 2.91]
**Subtotal (95% CI)**		**79**		**66**	**79.3%**	**1.31 [0.57, 3.01]**
Total events	12		7			
Heterogeneity: Tau² = 0.00; Chi² = 0.29, df = 1 (P = 0.59); I² = 0%						
Test for overall effect: Z = 0.63 (P = 0.53)						
**1.20.2 Post-mechanical ventilation extubation**						
Shin, 2022	4	35	2	35	20.7%	2.00 [0.39, 10.22]
**Subtotal (95% CI)**		**35**		**35**	**20.7%**	**2.00 [0.39, 10.22]**
Total events	4		2			
Heterogeneity: Not applicable						
Test for overall effect: Z = 0.83 (P = 0.40)						
**Total (95% CI)**		**114**		**101**	**100.0%**	**1.43 [0.68, 3.00]**
Total events	16		9			
Heterogeneity: Tau² = 0.00; Chi² = 0.50, df = 2 (P = 0.78); I² = 0%						
Test for overall effect: Z = 0.94 (P = 0.35)						
Test for subgroup differences: Chi² = 0.21, df = 1 (P = 0.65), I² = 0%						

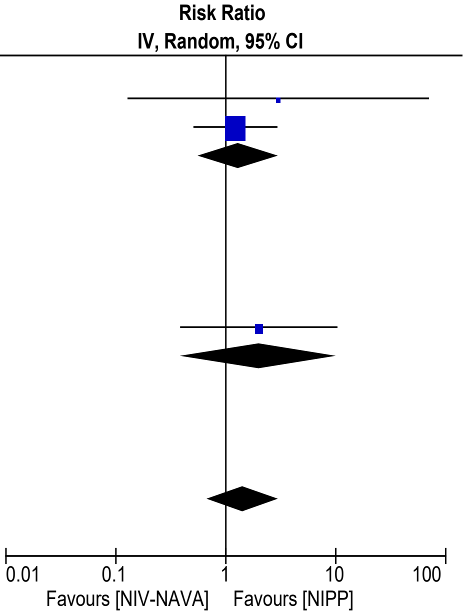

Supplementary Figure S7: Comparison Between NIV- NAVA Versus NCPAP, outcome "Mean time to full enteral feeding (days)"

	NIV-NAVA			NCPAP				Mean Difference
Study or Subgroup	Mean	SD	Total	Mean	SD	Total	Weight	IV, Random, 95% CI
**1.6.1 Primary ventilation mode**								
Kallio, 2019	4.9	2.4	20	5.3	2.3	20	93.7%	-0.40 [-1.86, 1.06]
**Subtotal (95% CI)**			**20**			**20**	**93.7%**	**-0.40 [-1.86, 1.06]**
Heterogeneity: Not applicable								
Test for overall effect: Z = 0.54 (P = 0.59)								
**1.6.2 Post-mechanical ventilation extubation**								
Shin, 2022	20	11.59	35	17	15.46	35	6.3%	3.00 [-3.40, 9.40]
**Subtotal (95% CI)**			**35**			**35**	**6.3%**	**3.00 [-3.40, 9.40]**
Heterogeneity: Not applicable								
Test for overall effect: Z = 0.92 (P = 0.36)								
**Total (95% CI)**			**55**			**55**	**100.0%**	**-0.19 [-1.80, 1.43]**
Heterogeneity: Tau² = 0.17; Chi² = 1.03, df = 1 (P = 0.31); I² = 3%								
Test for overall effect: Z = 0.23 (P = 0.82)								
Test for subgroup differences: Chi² = 1.03, df = 1 (P = 0.31), I² = 2.9%								

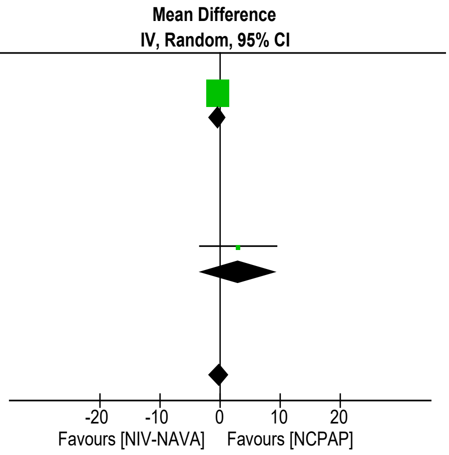

Supplementary Figure S8: Comparison Between NIV- NAVA Versus NCPAP, outcome "Mean pCO2"

	NIV-NAVA			NCPAP				Std. Mean Difference
Study or Subgroup	Mean	SD	Total	Mean	SD	Total	Weight	IV, Random, 95% CI
**1.30.1 Primary ventilation mode**								
Kallio, 2019	5.8	1	20	6.2	1	20	36.0%	-0.39 [-1.02, 0.23]
**Subtotal (95% CI)**			**20**			**20**	**36.0%**	**-0.39 [-1.02, 0.23]**
Heterogeneity: Not applicable								
Test for overall effect: Z = 1.23 (P = 0.22)								
**1.30.2 Post-mechanical ventilation extubation**								
Shin, 2022	44.5	7.65	35	46.4	10.9	35	64.0%	-0.20 [-0.67, 0.27]
**Subtotal (95% CI)**			**35**			**35**	**64.0%**	**-0.20 [-0.67, 0.27]**
Heterogeneity: Not applicable								
Test for overall effect: Z = 0.83 (P = 0.41)								
**Total (95% CI)**			**55**			**55**	**100.0%**	**-0.27 [-0.64, 0.11]**
Heterogeneity: Tau² = 0.00; Chi² = 0.23, df = 1 (P = 0.63); I² = 0%								
Test for overall effect: Z = 1.40 (P = 0.16)								
Test for subgroup differences: Chi² = 0.23, df = 1 (P = 0.63), I² = 0%								

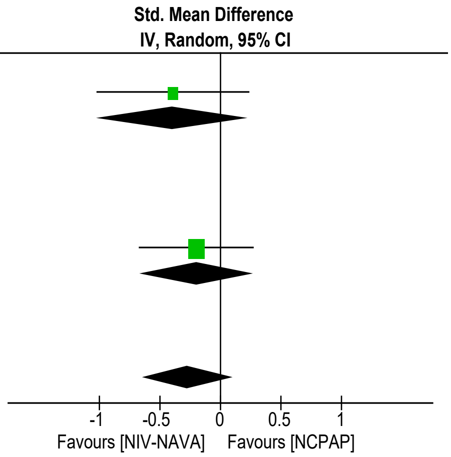

Supplementary Figure S9: Comparison Between NIV- NAVA Versus NCPAP, outcome "Mean pH"

	NIV-NAVA			NCPAP				Mean Difference
Study or Subgroup	Mean	SD	Total	Mean	SD	Total	Weight	IV, Random, 95% CI
**1.29.1 Primary ventilation mode**								
Kallio, 2019	7.33	0.7	20	7.31	0.05	20	1.3%	0.02 [-0.29, 0.33]
**Subtotal (95% CI)**			**20**			**20**	**1.3%**	**0.02 [-0.29, 0.33]**
Heterogeneity: Not applicable								
Test for overall effect: Z = 0.13 (P = 0.90)								
**1.29.2 Post-mechanical ventilation extubation**								
Shin, 2022	7.33	0.08	35	7.32	0.07	35	98.7%	0.01 [-0.03, 0.05]
**Subtotal (95% CI)**			**35**			**35**	**98.7%**	**0.01 [-0.03, 0.05]**
Heterogeneity: Not applicable								
Test for overall effect: Z = 0.56 (P = 0.58)								
**Total (95% CI)**			**55**			**55**	**100.0%**	**0.01 [-0.02, 0.05]**

Heterogeneity: Tau² = 0.00; Chi² = 0.00, df = 1 (P = 0.95); I² = 0%
Test for overall effect: Z = 0.57 (P = 0.57)
Test for subgroup differences: Chi² = 0.00, df = 1 (P = 0.95), I² = 0%

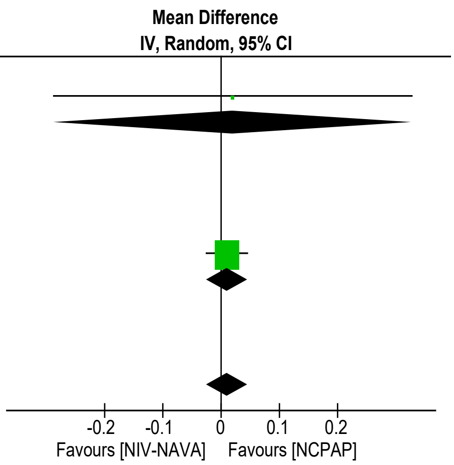

Supplementary Figure S10: Comparison Between NIV- NAVA Versus NIPP, outcome "Respiratory rate (RR)"

	NIV-NAVA			NIPP				Mean Difference	Risk of Bias					
Study or Subgroup	Mean	SD	Total	Mean	SD	Total	Weight	IV, Random, 95% CI	A	B	C	D	E	F
**1.10.1 Post-mechanical ventilation extubation**														
Gibu, 2017	67.5	14.9	8	66.8	14.1	8	36.2%	0.70 [−13.52, 14.92]	+	+	+	+	+	+
Treussart, 2022	55	13.18	14	52	15.65	14	63.8%	3.00 [−7.72, 13.72]	+	+	+	+	+	+
**Subtotal (95% CI)**			**22**			**22**	**100.0%**	**2.17 [−6.39, 10.72]**						
Heterogeneity: Tau² = 0.00; Chi² = 0.06, df = 1 (P = 0.80); I² = 0%														
Test for overall effect: Z = 0.50 (P = 0.62)														
**Total (95% CI)**			**22**			**22**	**100.0%**	**2.17 [−6.39, 10.72]**						

Heterogeneity: Tau² = 0.00; Chi² = 0.06, df = 1 (P = 0.80); I² = 0%
Test for overall effect: Z = 0.50 (P = 0.62)
Test for subgroup differences: Not applicable

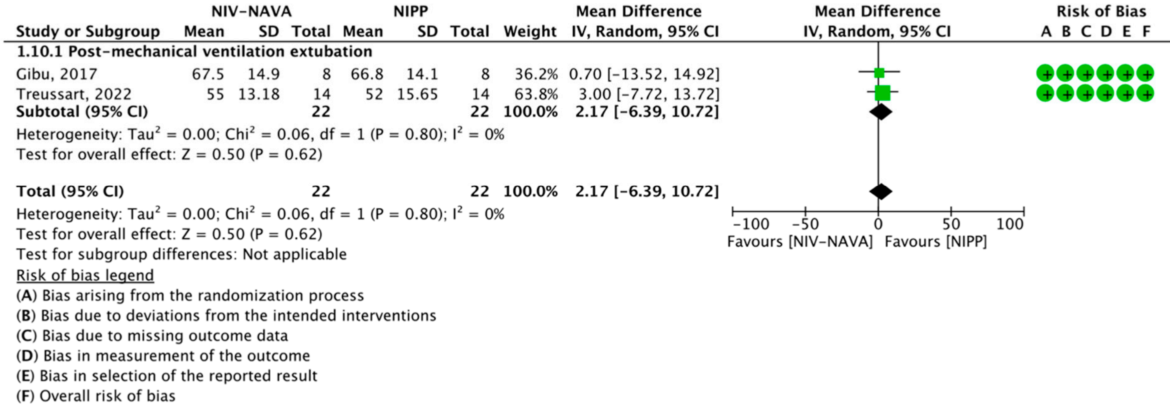

<u>Risk of bias legend</u>
(**A**) Bias arising from the randomization process
(**B**) Bias due to deviations from the intended interventions
(**C**) Bias due to missing outcome data
(**D**) Bias in measurement of the outcome
(**E**) Bias in selection of the reported result
(**F**) Overall risk of bias

Supplementary Figure S11: Comparison Between NIV- NAVA Versus NCPAP, outcome "Fraction of inspired oxygen (FiO_2)"

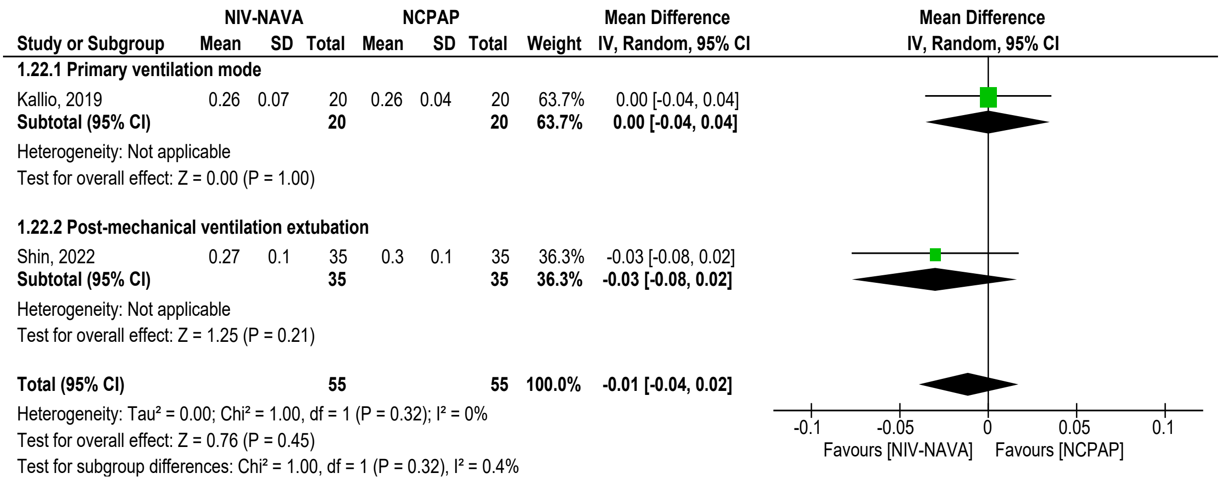

Supplementary Figure S12: Comparison Between NIV- NAVA Versus NIPP, outcome "Fraction of inspired oxygen (FiO_2)"

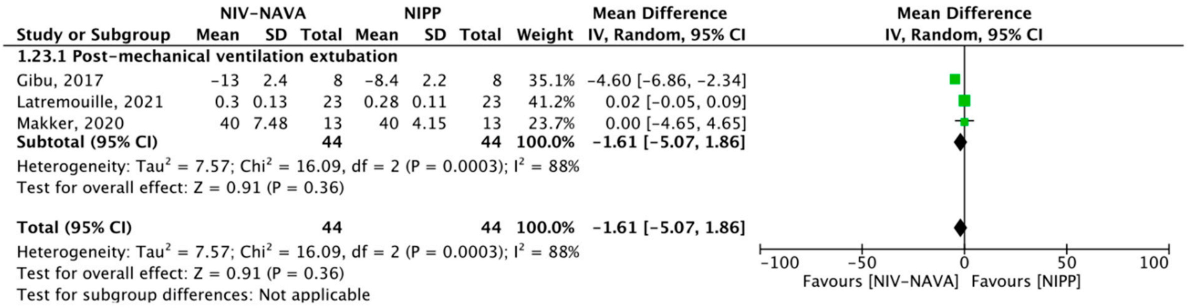

References:

1. Raju TN, Higgins RD, Stark AR, Leveno KJ. Optimizing care and outcome for late-preterm (near-term) infants: a summary of the workshop sponsored by the National Institute of Child Health and Human Development. *Pediatrics.* 2006; 118(3):1207-14.
2. Bolisetty S, Legge N, Bajuk B, Lui K. Preterm infant outcomes in New South Wales and the Australian Capital Territory. *Journal of Paediatrics and Child Health* 2015;**51**(7):713-21. [DOI: 10.1111/ jpc.12848; PUBMED: 25644196]
3. Darnall RA. The role of CO(2) and central chemoreception in the control of breathing in the fetus and the neonate. *Respiratory Physiology & Neurobiology* 2010;**173**(3):201-12. [DOI: 10.1016/ j.resp.2010.04.009; PUBMED: 10.1016/j.resp.2010.04.009]
4. Beck J, Brander L, Slutsky AS, Reilly MC, Dunn MS, Sinderby C. Non-invasive neurally adjusted ventilatory assist in rabbits with acute lung injury. *Intensive Care Medicine* 2008;**34**(2):316-23. [DOI: 10.1007/s00134-007-0882-x; PUBMED: 17960364]
